# Effectiveness of a nursing intervention in decreasing the anxiety levels of family members of patients undergoing cardiac surgery: a randomized clinical trial

**DOI:** 10.1590/1518-8345.0208.2729

**Published:** 2016-08-15

**Authors:** Letícia Hamester, Emiliane Nogueira de Souza, Cibele Cielo, Maria Antonieta Moraes, Lúcia Campos Pellanda

**Affiliations:** 1 Student of the Primary Health Care Residency Program - Public Health, Escola de Saúde Pública do Rio Grande do Sul, Porto Alegre, RS, Brazil.; 2 PhD, Professor, Departamento de Enfermagem, Universidade Federal de Ciências da Saúde de Porto Alegre, Porto Alegre, RS, Brazil.; 3 Student of the Multidisciplinary Integrated Health Residency Program - Nursing in Cardiology, Instituto de Cardiologia, Fundação Universitária de Cardiologia, Porto Alegre, RS, Brazil.; 4 PhD, Professor, Instituto de Cardiologia, Fundação Universitária de Cardiologia, Porto Alegre, RS, Brazil.; 5 PhD, Professor, Departamento de Saúde Coletiva, Universidade Federal de Ciências da Saúde de Porto Alegre, Porto Alegre, RS, Brazil.

**Keywords:** Orientation, Perioperative Nursing, Professional-Family Relations, Thoracic

## Abstract

**Objective:**

to verify the effectiveness of nursing orientation provided to families of patients in the immediate post-operative following cardiac surgery before the first visit to the post-anesthesia care unit, in decreasing anxiety levels, compared to the unit's routine orientation.

**Method:**

open randomized clinical trial addressing family members in the waiting room before the first visit in the immediate post-operative period. The family members assigned to the intervention group received audiovisual orientation concerning the patients' conditions at the time and the control group received the unit's routine orientation. Outcome anxiety was assessed using the STAI-State.

**Results:**

210 individuals were included, 105 in each group, aged 46.4 years old on average (±14.5); 69% were female and 41% were the patients' children. The mean score obtained on the anxiety assessment in the intervention group was 41.3±8.6, while the control group scored 50.6±9.4 (p<0.001).

**Conclusion:**

a nursing intervention focused on providing guidance to families before their first visit to patients in the immediate post-operative period of cardiac surgery helps to decrease the levels of anxiety of companions, making them feel better prepared for the moment. ReBEC (Brazilian Clinical Trials Registry) and The Universal Trial Number (UTN), No. U1111-1145-6172.

## Introduction

The perioperative period of cardiac surgeries exposes patients and families to feelings and emotions that make them experience much stress and anxiety. Anxiety is considered a cognitive, affective and behavioral disorder, caused by situations that are perceived as threatening^1^. The desire to obtain information and the expectation to visit a family member in a hospital setting may aggravate the perceptions of family members, regardless of the surgical procedure.

In this context, providing information regarding the perioperative period and alleviating the anxiety of surgical patients has been the focus of nursing care. Care provided to family members, however, also demands attention. The hospitalization, surgical procedure and recovery in an intensive care unit cause anxiety because the family has to face an unknown situation the patient is inserted in that is full of uncertainty^2^ ^-^ ^3^. Note that anxiety is at highest when family members are, most often, in waiting rooms, awaiting information regarding the surgical intervention^1^. The contact with a hospital setting and the expectation of meeting the patient surrounded by monitoring equipment cause doubts and anxiety in family members^4^. 

The inclusion of family members in perioperative orientation can help the family to feel safer about the impending surgery. When well oriented, family members encourage the patient and help in the recovery. Considering that changes affecting one family member potentially affect the remaining members of the family, interventions directed to the entire family can indirectly benefit the patient^5^. For this reason, the establishment of an effective relationship between the health staff and family members minimizes insecurity and distress, ultimately contributing to overcome difficulties^2^ ^,^ ^4^. 

The literature shows that visiting a patient in the recovery room only is not sufficient to significantly reduce the anxiety of family members^6^. Interventions implemented at the time the family is waiting to visit the patient in the Immediate Post-Operative (IPO) period have been tested, aiming to decrease anxiety levels among family members. A pre-post intervention study, conducted to verify the effect of watching a movie in the waiting room, reports decreased anxiety levels among the family members of surgical patients (from 46±9.2 to 39.1±11.8 points; p=0.003)^7^. Another study, conducted to verify differences in the levels of anxiety, stress and relaxation of family members in the waiting room of a surgical center after listening to live music for 20 minutes, show that even though the outcome levels did not significantly decrease after the intervention, relaxation scores increased (p=0.0008)^8^. Nonetheless, strategies to encourage greater interaction between the nursing staff and family members while in the waiting room, including intergroup comparisons, have not been tested yet.

Given the previous discussion, this study's objective was to verify the effectiveness of nursing guidance provided to the families of patients in the immediate post-operatory period of cardiac surgery before the first visit in the post-anesthesia care unit in decreasing anxiety levels when compared to the unit's routine guidance.

## Method

This open randomized clinical trial was registered in the ReBEC (Brazilian Registry of Clinical Trials) and in the Universal Trial Number (UTN) under No. U1111-1145-6172, and conducted in a cardiac reference hospital in the South of Brazil from March to June 2013. 

The sample was composed of family members of patients undergoing CABG or heart valve surgery, who were expecting the patient to return from surgery in the waiting room across the surgical center and awaited the first visit in the Immediate Post-Operative Care Unit (IPO). Inclusion criteria were: both sexes, 18 years old or older, being a family member/caregiver, and consent to participate in the study. Those who had already accompanied a patient in prior cardiac surgery were excluded. The sample size was based on a previous study^5^, considering that 52.5% of the companions presented average levels of anxiety. We estimated that 105 family members would be necessary in each group to obtain a difference of 20% in the average level of anxiety, with power of 80% and 95% of confidence level. 

Family members were approached in the IPO's waiting room after being invited to participate, receiving clarification, and signing free and informed consent forms, and were assigned to the Intervention Group (IG) or Control Group (CG), using simple random sampling obtained at http://www.randomizer.org, established for one week: the individuals approached in week 1 were assigned to IG and those in week 2 were assigned to the CG. An independent professional performed the randomization. Both groups answered a structured questionnaire, addressing sociodemographic variables and variables related to preparation to visit a patient in the IPO.

The intervention was implemented by one of the researchers, a nurse with experience in the immediate post-operative following cardiac surgery, and was composed of audiovisual orientation, provided in groups in the period prior to the visit. The family members were oriented in the waiting room about the potential conditions the patient would face post cardiac surgery, using audiovisual resources such as illustrative images of equipment/devices used in the immediate post-operatory. The intervention lasted approximately 20 minutes, after which the individuals were encouraged to clarify doubts. The CG received orientation routinely provided in the unit, which includes aspects related to the sector's routine, such as visiting hours, hand washing, maximum number of visitors, and the fact that the medical staff would provide information regarding the patient's condition after visitation. A nursing technician, nurse or members of the surgical team usually provides such information.

The family could visit the patient at hours previously established by the unit. Visits lasted up to 30 minutes and only one visitor was allowed per time. The anxiety outcome was verified using the State-Trait Anxiety Inventory - STAI after intervention and before the first visit. 

STAI is a self-report questionnaire widely used in the monitoring of anxious states, composed of two distinct scales developed to measure two concepts of anxiety, trait and state anxiety. It has been translated and validated for the Brazilian population with satisfactory psychometric properties^9^. In this study, only the State Anxiety Inventory (STAI-State) was used because anxiety was assessed before the post-operatory visit, when the instrument was handed to the family member to complete and then return it to the researcher.

The concept of state anxiety is defined as a transitory emotional state, or as a condition of the human body characterized by unpleasant feelings of stress and tension, consciously perceived and accompanied by increased activity of the autonomous nervous system. In this self-reported 20-item tool, the intensity of anxiety is classified on a Likert scale that ranges from 1 (absolutely not) to 4 (very much). The total score ranges from 20 to 80, in which higher scores indicate higher levels of anxiety. Scores of questions with a positive nature are inverted, that is, if the individual answers 4, 1 is attributed in the coding process, if s/he answers 3, 2 is attributed, if answers 2, then 3 is attributed and, if the individual answers 1, 4 is attributed. Levels of anxiety were categorized for the purpose of analysis^5^: scores from 19 to 40 were considered to be low, from 41 to 60 - average, and scores from 61 to 76 were considered high.

The study received approval from the Institutional Review Board at the Cardiology Institute of Porto Alegre, RS, Brazil (CAAE 09904012.0.0000.5333). 

Data were analyzed using SPSS (Statistical Package for the Social Sciences), version 18.0. Categorical variables were expressed through absolute and relative frequencies and continuous variables were presented using mean and standard deviation, according to normal or non-normal distribution. The unpaired Student's *t* test was used for intergroup comparisons regarding the level of anxiety variable (numerical) and remaining quantitative variables. The Chi-square test was used to check for associations between qualitative variables and the group variable. The Mann-Whitney test was utilized to compare the groups with regard to the outcome (anxiety) when it was treated as an ordinal qualitative variable. A level of significance of 5% was considered. 

## Results

**Figure 1:**
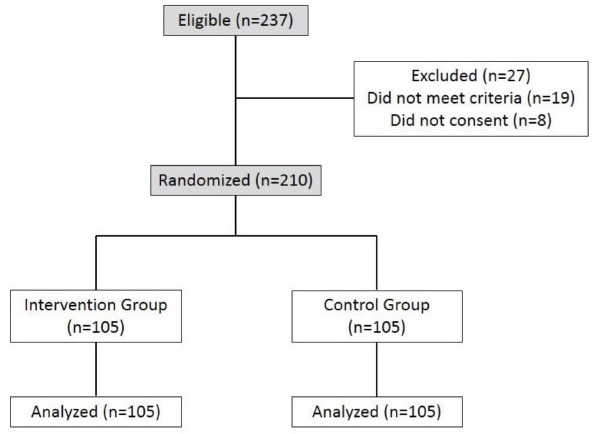
CONSORT Diagram. Porto Alegre, RS, Brazil, 2013

Figure 1 shows the allocation of the study's subjects.

A total of 210 relatives of patients in the immediate postoperative period after cardiac surgery participated in the study. The characterization of family members and patients is presented in Table 1. No statistical differences were found in the comparison between the groups' variables (p>0.05).

**Table 1:** Characterization of the sample of family members and patients. Porto Alegre, RS, Brazil, 2013

Variables			All (n=210)	IG* (n=105)	CG^†^ (n=105)
Family members					
	Age^‡^		46.4 (±14.5)	46.58 (±14.6)	46.2 (±14.5)
	Sex. women		145 (69.0)	74 (70.5)	71 (67.6)
	Education				
	Illiterate		9 (4.3)	6 (5.7)	3 (2.9)
	Up to middle school		66 (31.4)	35 (33.3)	31 (29.5)
	Up to high school		59 (28.1)	30 (28.6)	29 (27.6)
	Up to college		76 (36.2)	34 (32.4)	42 (40.0)
	Kinship with patient				
		Son	86 (41.0)	39 (37.1)	47 (44.8)
		Spouse	51 (24.3)	29 (27.6)	22 (21.0)
		Brother	14 (6.7)	8 (7.6)	6 (5.7)
		Son/daughter-in-law	14 (6.7)	7 (6.6)	7 (6.6)
		Others	45 (21.4)	22 (20.9)	23 (21.9)
	Prior information about POI visit^§^		46 (21.9)	26 (24.7)	20 (19.0)
Patients					
	Age^‡^		62.1 (±11.5)	60.8 (±11.4)	63.5 (±11.6)
	Sex, men		117 (55.7)	59 (56.1)	58 (55.2)
	Type of surgery				
		CABG^||^	134 (63.8)	61 (58.1)	73 (69.5)
		Heart valve surgery	76 (36.2)	44 (41.9)	32 (30.4)

*IG: Intervention Group; †CG: Control Group; ‡Variable presented in mean and standard deviation; §IPO: Immediate Post-Operative; ||CABG: Coronary artery bypass grafting.

In response to the question asked after the intervention "how do you feel about visiting your relative in the post-operatory?", in total, 63.3% of the individuals reported feeling prepared and confident. A significantly higher proportion of the IG, however, reported feeling prepared and confident before first visiting the patient in the IPO (IG 82;78.1% vs. CG 51;48.6%; p<0.001), as presented in Table 2.

**Table 2:** Comparison between the answers of both groups regarding how they felt before visiting their relatives in the post-operatory unit. Porto Alegre, RS, Brazil, 2013

Answers	All (n=210)	IG* (n=105)	CG^†^ (n=105)	p^‡^
Prepared and confident	133 (63.3%)	82 (78.1%)	51 (48.6%)	<0.001
Prepared but afraid	42 (20%)	17 (16.2%)	25 (23.8%)	
Unprepared but confident	28 (13.3%)	3 (2.9%)	25 (23.8%)	
Unprepared and afraid	7 (3.3%)	3 (2.9%)	4 (3.8%)	

*IG: Intervention Group; †CG: Control Group; ‡ Mann Whitney test to compare between groups

The average score obtained in the anxiety assessment by the IG was 41.3±8.6 and by the CG was 50.6±9.4 (p<0.001). The distribution of the sample among levels of anxiety is shown in Table 3.

**Table 3:** Comparison among levels of anxiety between the groups according to STAI -State. Porto Alegre, RS, Brazil, 2013

Anxiety levels	Total (n=210)	IG* (n=105)	CC^†^ (n=105)	p^‡^
Low	15 (7.1%)	12 (11.4%)	3 (2.9%)	<0.001
Intermediate	114 (54.3%)	75 (71.4%)	39 (37.1%)	
High	81 (38.6%)	18 (17.1%)	63 (60%)	

*IG: Intervention Group; †CG: Control Group; ‡ Mann Whitney test to compare between groups

The individuals were given an opportunity to clarify doubts after the intervention and the researchers answered the questions. The main doubts concerning the IPO period included: the unit's routine, length of stay in the unit and hospital, what could possibly happen if the patient became too emotional when seeing family, and questions concerning how long the patient should wait before resuming daily activities. 

## Discussion

This randomized clinical trail addressing the relatives of patients undergoing cardiac surgery shows that nursing orientation provided at the time preceding the first visit in the immediate post-operatory unit contributed to decrease anxiety levels of family members and helped them to feel prepared to visit the patient in the post-anesthesia care unit. 

The STAI-State assessment revealed significant decrease in the anxiety levels of individuals in the IG when compared to those from the CG. Most individuals from the CG presented high levels of anxiety, while those in the IG remained at intermediary levels. The moment that antecedes the first visit in the immediate post-operatory unit generates anxiety that cannot be completely eliminated in the face of an unknown situation involving a family member, though comfort and reassurance can be provided during the waiting time, which help alleviate tension.

A study conducted with family members and patients undergoing coronary angiography for the first time, aiming to identify levels of anxiety during the waiting period for the exam, verified that the mean score the patients' companions obtained in the STAI-State was 42.8±9.9, while intermediate levels of anxiety predominated among most companions (52.5%)^5^. In this study, the mean score the IG obtained in the STAI-State after intervention was similar, while the CG obtained higher scores (41.3±8.6 versus 50.6±9.4; p<0.001).

Another study^10^, addressing 41 family members, verified the perception of individuals concerning visitation to the intensive care unit and found that the feeling most frequently mentioned at the time of visitation was anxiety, followed by distress and sorrow. It is known that the visit in the immediate post-operatory period contributes to decrease the anxiety of family members of patients who underwent a cardiac surgery^11^. Fear and insecurity, arising from uncertainty concerning the patient's conditions after surgery, however, may be intensified if family members are not properly informed about the situation. Such a fact might explain the findings of a study conducted in Greece with the family members of surgical patients, aiming to determine whether the visit to patients in the post-anesthetic care unit in the post-operatory period would decrease the anxiety of family members. The results showed that scores obtained in the STAI-State were higher before than after the visit (57 [23-80] versus 51 [21-77]; p=0.000008). The visit, however, did not significantly decrease anxiety levels measured before and after the visit (from 76% to 58%, respectively)^6^.

These feelings become more intense when facing a cardiac surgery, the impact of which is considerable, not only on patients but also on family members. Hence, care and support should be offered to family members as they closely share the distress that involves the perioperative period^12^. In addition to providing printed pamphlets containing information regarding how long the procedure lasts, informing the recovery room's routine and useful telephone numbers, verbal interaction with a nurse from the sector where the patient will recover can also minimize the anxiety of those waiting for news and an opportunity to visit the patient^13^. Additionally, the mere fact of feeling properly treated by the service already minimizes the feeling of helplessness and, as a consequence, anxiety.

When asked about how they felt before visiting the patient for the first time after surgery, most family members from the IG answered "prepared and confident", while less than half of the CG provided a positive answer. This finding shows that orientation provided during this study's intervention contributed for family members to feel more prepared, whether because of the nursing guidance provided or because of the reception they received. The onset of a severe disease and the distress experienced by the family cannot be prevented. Nonetheless, identifying the needs of families and planning interventions that meet the real demands of both patient and family are essential for the work of the nursing staff ^(^ ^3^.

Orientations provided to family members before the first visit in the immediate post-operatory period are rare, leaving these individuals unprepared as to how to behave towards the patient, what care is provided, and regarding the sector's dynamics. This lack of preparation can cause emotional stress that negatively affects the visit. The interaction between professionals and family members before the visit can alleviate stress, a feeling that is common in this period, allowing the family to feel reassured^10^ ^,^ ^14^. One study aiming to determine the needs and experiences of patients and families in the perioperatory period reports that the highest scores obtained by the family members included in the study referred to the need to communicate with the surgeon after the procedure, the opportunity to ask questions, and have concerns clarified by the hospital's staff, in addition to having information regarding the surgical procedure per se^15^. The intervention conducted in this study is one strategy that can be used to meet these needs because it enables interaction between family members and IPO nurses.

In another study, the objective of which was to identify orientations nurses provided to the family members of patients hospitalized in an ICU at the time of visitation, found that 52.6% of the nurses provided orientation during the first visit; 36.8% reported they did not provide any orientation; and 10.5% reported that orientation was provided depending on the situation^16^. There is a concern on the part of nurses to provide orientation to family members during the first visit, while clarifying norms and routines and reporting the condition of patients are among the individuals' main needs. There are, however, different demands workers accompanying the patient and family need to meet in order to establish a relationship of trust from the time the patient is admitted. The supply of information improves the bond established between patient and institution, and with professionals, consequently decreasing anxiety concerning the hospitalization period, surgery, anesthesia and hospital routines, among other common doubts^17^.

The communication process is key in the care provided to families. The attitudes of family members can change after being properly oriented; the family becomes more cooperative and confident toward the care provided. The nursing staff should meet these needs and establish greater interaction with families, also perceiving them as clients who demand care, so that integral care is provided, minimizing anxiety through the establishment of trust, cooperation, understanding, acceptance of diagnosis and procedures used, improving understanding and empathy, enabling family members to take part in decision making regarding care delivery^18^ ^-^ ^19^. 

Not only the patient, but also those accompanying the patient should receive care. We should only allow the family member to visit the patient after surgery; families need to be prepared for the moment. It is known that the waiting period is tense and distressing. Small actions on the part of the staff may go a long way. Situations that for healthcare workers are commonplace, such as the monitoring of patients with sound alarms and other devices, can be threatening for a concerned relative who waited hours to see a patient. A lack of information can be interpreted as something more serious than it really is. A family member who feels prepared and confident for the first visit can better grasp the staff's work process and is not easily scared by seeing the handling of equipment; on the contrary, (s)he is able to understand a patient's real needs, most of which are temporary, and to collaborate with the care provided.

This study's limitations include the fact that anxiety was not assessed in the pre-intervention period (baseline) because the family members usually arrived at the waiting room approximately 30 minutes before visitation. Additionally, family members accompanying patients for the first time were not compared with those who had this experience. Therefore, there is a need to apply a statistical model considering potential confounding variables. 

## Conclusion

A nursing intervention focused on providing guidance to family members at the time that antecedes the first visit in the IPO unit of cardiac surgery helps decreasing the anxiety of family members, making them feel more prepared for the visit, so that it is more useful and beneficial to both families and patients.

Note that this is a low cost intervention that can be implemented in other contexts of waiting rooms for surgical patients. We recommend, however, that further studies with experimental designs, including pre- and post-intervention, be conducted to confirm the results of this study.

Nursing interventions focused on family members implemented in health care services humanize care delivery, strengthen the bonds between professionals and families and contribute to the recovery of patients. As there are not always professionals available and some are not properly prepared to provide information and interact with families, we suggest, as a complementary strategy, to develop and distribute educational brochures to clarify doubts. 

This study contributes to the implementation of communication strategies directed to family members of patients in the immediate post-operatory period, intended to improve the nurse/patient/family relationship in line with the recovery process of patients.

## References

[1] Munday J, Kynoch K, Hines S. (2013). The effectiveness of information-sharing interventions as a means to
reduce anxiety in families waiting for surgical patients undergoing an elective
surgical procedure: a systematic review protocol. JBI Library.

[2] Beuter M, Brondani CM, Szareski C, Cordeiro FR, Castro C (2012). Sentimentos de familiares acompanhantes de adultos face ao processo de
hospitalização. Esc Anna Nery.

[3] Freitas KS, Kimura M, Ferreira KASL (2007). Family members' needs at intensive care units comparative analysis
between a public and a private hospital. Rev. Latino-Am. Enfermagem.

[4] Maestri E, Nascimento ERP, Bertoncello KCG, Martins JJ (2012). Avaliação das estratégias de acolhimento na Unidade de Terapia
Intensiva. Rev Esc Enferm USP.

[5] Grazziano ES, Bianchi ERF (2004). Caregivers and patient's anxiety level during cardiac catheterizathion
Rev. Latino-Am. Enfermagem.

[6] Patelarou A, Melidoniotis E, Sgouraki M, Karatzi M, Souvatzis X (2014). The effect of visiting surgical patients in the postanesthesia care
unit on family members' anxiety a prospective quasi-experimental
study. J Perianesth Nurs.

[7] Mojdeh S, Zamani M, Kooshki AM, Jafari N (2013). Effect of watching a movie on family members' anxiety level during
their relatives' surgery. Iran J Nurs Midwifery Res.

[8] Jarred Jennifer D. (2003). The effect of live music on anxiety levels of persons waiting in a surgical
waiting room as measured by self-report.

[9] Biaggio AMB, Natalcio L, Spielberger CD (1977). Desenvolvimento da forma experimental em português do
IDATE. Arq Bras Psicol.

[10] Beccaria LM, Ribeiro R, Souza GL, Scarpetti N, Contrin LM, Pereira RAM (2008). Visita em unidades de terapia intensiva concepção dos familiares
quanto à humanização do atendimento. Arq Ciênc Saúde.

[11] Trecartin K, Carroll DL (2011). Nursing information for family members waiting during cardiac
procedurs. Clin Nurs Res.

[12] Palmeira CG, Peralva ELM, Batista FQ. (2007). A importância da oferta de suporte psicológico aos familiares de
pacientes submetidos à cirurgia cardíaca. Rev Bras Cardiol.

[13] Muldoon M, Cheng D, Vish N, Dejong S, Adams J (2011). Implementation of an informational card to reduce family members'
anxiety. AORN J.

[14] Méllo DC, Rodrigues BMRD (2008). O acompanhante de criança submetida à cirurgia cardíaca contribuição
para a enfermagem. Esc Anna Nery.

[15] Davis Y, Perham M, Hurd AM, Jagersky R, Gorman WJ, Lynch-Carlson D (2014). Patient and family member needs during the perioperative
period. J Perianesth Nurs.

[16] Silva ND, Cotrin LM (2007). Orientações do enfermeiro dirigidas aos familiares dos pacientes
internados na UTI no momento da visita. Arq Ciênc Saúde.

[17] Carvalho ACS, Lacerda AC. (2010). A Enfermagem atuando na educação de pacientes e familiares: uma visão
ampliada. Rev Pesq Cuid Fundam.

[18] Filho WDL, Nunes AC, Pauletti G, Lunardi VL (2004). As manifestações de ansiedade em familiares de pacientes internados em
unidades de terapia intensiva gerais. Fam Saúde Desenv.

[19] Soares M (2007). Cuidando da família de pacientes em situação de terminalidade
internados na unidade de terapia intensiva. Rev Bras Ter Intensiva.

